# Unravelling mysteries at the perivascular space: a new rationale for cerebral malaria pathogenesis

**DOI:** 10.1016/j.pt.2023.11.005

**Published:** 2023-12-08

**Authors:** Samuel C. Wassmer, Tania F. de Koning-Ward, Georges E.R. Grau, Saparna Pai

**Affiliations:** 1 Department of Infection Biology, London School of Hygiene & Tropical Medicine, London, UK; 2 School of Medicine, Deakin University, Waurn Ponds, Victoria, Australia; 3 Institute of Mental and Physical Health and Clinical Translation, Deakin University, Geelong, Victoria, Australia; 4 Vascular Immunology Unit, Discipline of Pathology, School of Medical Sciences, University of Sydney, Camperdown, New South Wales, Australia; 5 Centre for Molecular Therapeutics, Australian Institute of Tropical Health and Medicine, James Cook University, Cairns, Queensland, Australia 6; 6 These authors contributed equally to the work.; 7 Lead contact.

## Abstract

Cerebral malaria (CM) is a severe neurological complication caused by *Plasmodium falciparum* parasites; it is characterized by the sequestration of infected red blood cells within the cerebral microvasculature. New findings, combined with a better understanding of the central nervous system (CNS) barriers, have provided greater insight into the players and events involved in CM, including site-specific T cell responses in the human brain. Here, we review the updated roles of innate and adaptive immune responses in CM, with a focus on the role of the perivascular macrophage–endothelium unit in antigen presentation, in the vascular and perivascular compartments. We suggest that these events may be pivotal in the development of CM.

## Redefining CM

Severe malaria is caused predominantly by *Plasmodium falciparum* parasites [1]. One of its clinical manifestations is CM, which takes a significant toll on human life each year [2]. Like many diseases that affect the **central nervous system** (CNS) (see Glossary), CM is characterized by cerebrovascular dysfunction. A dynamic, coordinated interplay between blood vessels, neurons, and resident immune cells is essential to brain health, and there is evidence that a dysregulation of these interactions is responsible for CM [3]. Typically, its neuropathology results from **cytoadherence** of *P*. *falciparum*-infected red blood cells (iRBCs) to the **endothelium**, leading to a phenomenon defined as sequestration [4]. However, this paradigm has undergone significant refinement in the past few years owing to several advances made in the field. For example, single-cell genomic technology can now functionally segregate brain arteries, veins, and capillaries [5]. Intravital microscopy has enabled visualization of parasites and immune cell activity in the brain in real time [6–8]. Discovery of the **glymphatic system** has set the stage for understanding how immune cells are activated by CNS antigens [9]. Moreover, increased availability of magnetic resonance imaging (MRI) facilities in malaria-endemic countries, new screening, diagnostic and prognostic biomarkers, as well as emerging adjunctive therapeutic options [10] have allowed a leap forward in our understanding, identification, and treatment of the disease. We present this review against a backdrop of such recent developments, and highlight new hypotheses for the pathogenesis of CM.

Over the years, evidence derived from human and experimental mouse models of CM (HCM and ECM, respectively) has mounted to support a constellation of inter-related pathophysiologic mechanisms in CM, which goes beyond parasite sequestration. Here, we discuss evidence showing that CM is a multifactorial syndrome involving innate and adaptive immune cell activation, antigen presentation at the blood–brain barrier (BBB), release of proinflammatory mediators, endothelial dysfunction, and BBB disruption. Reviewing the relevance of observations made in rodent models to human disease is timely. We present a selection of studies that used innovative tools to identify the role of parasite factors, endothelium, perivascular **myeloid cells** and antigen presentation in CM. The role of these factors in providing a vascular niche for the recruitment and activation of immune cells, release of cytotoxic mediators, and BBB disruption, is reviewed.

## Role of RBC sequestration in CM

**Figure 1.**
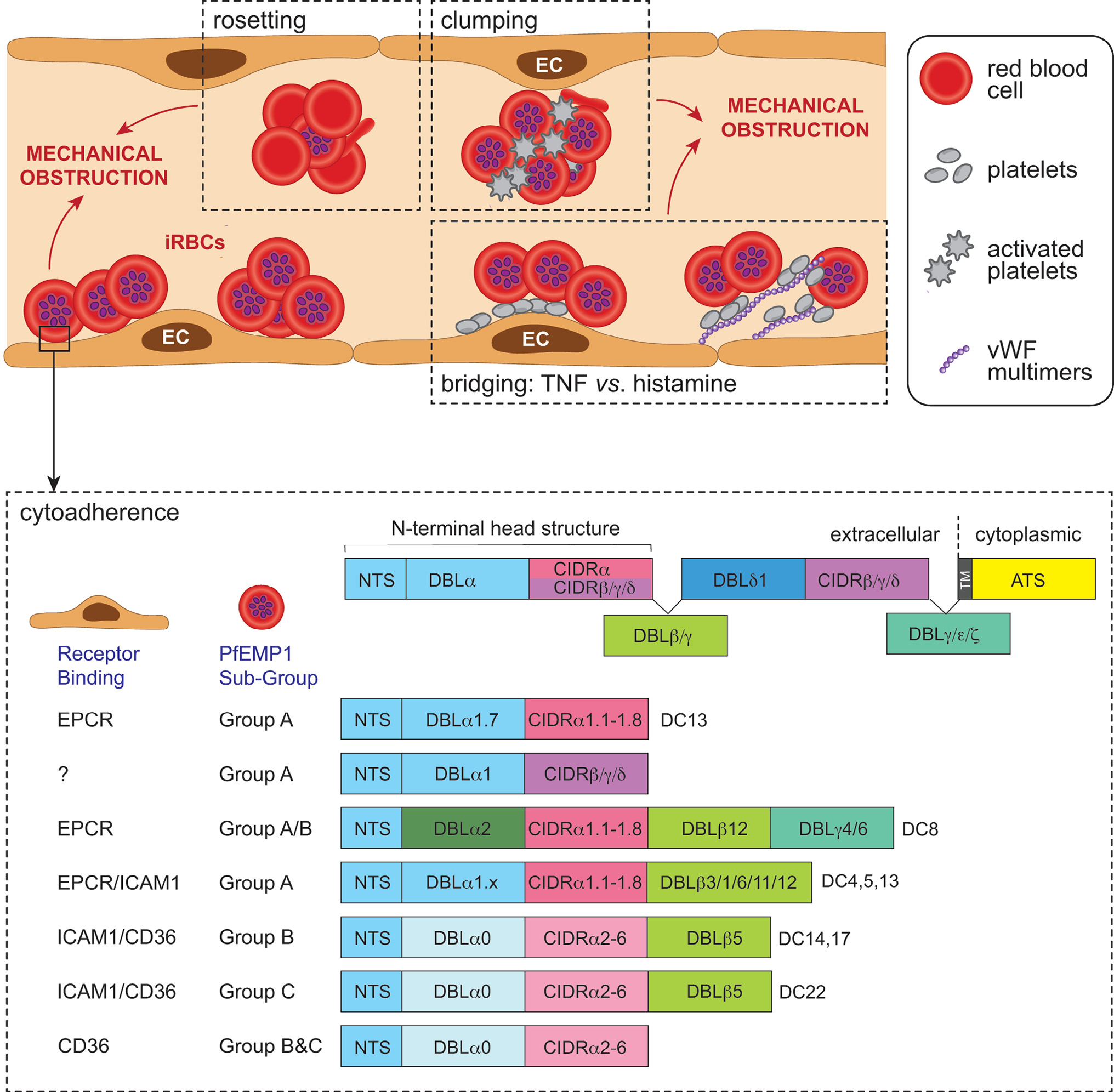
Schematic representation of the sequestration of *Plasmodium falciparum*-infected red blood cells (iRBCs) in cerebral malaria (CM). The expression of *P. falciparum*-infected erythrocyte membrane protein 1 (PfEMP1) on the iRBC surface leads to sequestration of the iRBC. In addition to the direct sequestration of iRBCs, additional mechanisms contribute to cerebral microvessel plugging and ischemic injury during CM. These include rosetting, the binding of uninfected erythrocytes to iRBCs to avoid immune clearance, and clumping of platelets and iRBCs. Platelets can also bridge CD36-binding PfEMP1 and tumour necrosis factor- (TNF)-activated endothelial cells (ECs); or iRBC and von Willebrand factor (vWF) multimers produced by histamine-activated endothelium. The PfEMP1 antigens are encoded by one of 60 *var* genes; one PfEMP1 variant is expressed by a single iRBC at any one time. The extracellular region of PfEMP1 is encoded by Duffy-binding-like domains (DBL) and cysteine-rich interdomain regions (CIDR); their particular combination in PfEMP1 variants has led to classification of domain cassettes (DCs) that enable binding to diverse receptors, including intercellular adhesion molecule 1 (ICAM-1), endothelial protein C receptor (EPCR), CD36 and oncofetal chondroitin sulfate A (CSA) expressed on ECs. ICAM-1 and EPCR are expressed in the brain, but there is either little or no constitutive CD36. EPCR binding occurs via the CIDRα1 domain, CD36 binding via the CIDRα2–6 domain, and ICAM binding may occur via DBLβ domains, while the binding phenotype of CIDRβ,γ,ε is unknown. PfEMP1 proteins are also classified as Group A, A/B, B or C according to their chromosomal location, upstream promoter sequence, and direction of *var* gene transcription. Group A and A/B are linked with severe disease, including CM, and they comprise subclasses of DBL and CIDR domains that bind EPCR or ICAM1. Group A EPCR or dual EPCR/ICAM-1 binders are most clearly associated with CM (adapted from [120]).

The sequestration of iRBCs within the cerebral vasculature is a hallmark of HCM in both adults and children (Table 1). Descriptions of iRBCs sequestered in capillaries and post-capillary venules of the brain date back to 1894 and have long been suspected to be the main pathophysiologic event in HCM [11]. However, recent quantitative analyses of post-mortem samples revealed that iRBC sequestration in the brain also occurs in patients with severe malaria without HCM [12], suggesting that the phenomenon is necessary but not sufficient to trigger the neurological syndrome. Sequestration of *P. falciparum*-iRBCs is mediated by specific domains of the erythrocyte membrane protein 1 (PfEMP1) family [13] (Figure 1). However, PfEMP1 is not the only parasite ligand that enables the cerebral accumulation of iRBCs, which is also observed in the human malaria species *Plasmodium vivax*, and species that infect rodents do not express PfEMP1. These species can also cause CM, albeit rarely in vivax malaria. The ligands that mediate their binding to the endothelium remain unknown. The sequestration of iRBCs not only leads to a mechanical obstruction of blood flow in capillaries and post-capillary venules, but also contributes to endothelial activation and pathology (Figure 1). Parasite products released from sequestered iRBCs also play critical roles (Box 1). One of the released parasite molecules is *P. falciparum* histidine-rich protein 2, and its production is proportionate to iRBC numbers, making it a useful surrogate of parasite biomass in infected individuals. Brain capillaries and post-capillary venules are somehow more vulnerable to iRBC sequestration than those from other organs, and this can lead to congestion, hypoxia, tissue swelling, coma, and ultimately, death [12]. Beside the essential role of sequestration, susceptibility stems form a complex interplay between parasite and host factors, with endothelial dysfunction, dysregulation of coagulation, resident and infiltrating immune cell type and localization, as well as inflamed microenvironment, all contributing in varying degrees to iRBC sequestration and development of HCM [3].

## CM pathogenesis: more than meets the eye

Post-mortem examinations of the brain of Malawian children and Vietnamese adults who died of CM revealed that, in addition to iRBC sequestration, pathological abnormalities such as demyelination, axonal damage, glial activation, haemorrhage, retinopathy, brain swelling, and BBB disruption are also seen (Table 1), and not all of these anomalies can be solely linked to iRBC sequestration. Investigations into the cause of death in HCM using MRI have highlighted the role of severe brain swelling and **tonsillar herniation** due to vasogenic oedema in children [14,15], while global hypoxic injury linked to high levels of sequestration was reported in adults [14]. In this section, we review the contribution of host factors in the development of CM, while tempering the simplistic view of iRBC sequestration as the sole driver of pathogenesis.

### Inflammatory cascades in CM

**Figure 2.**
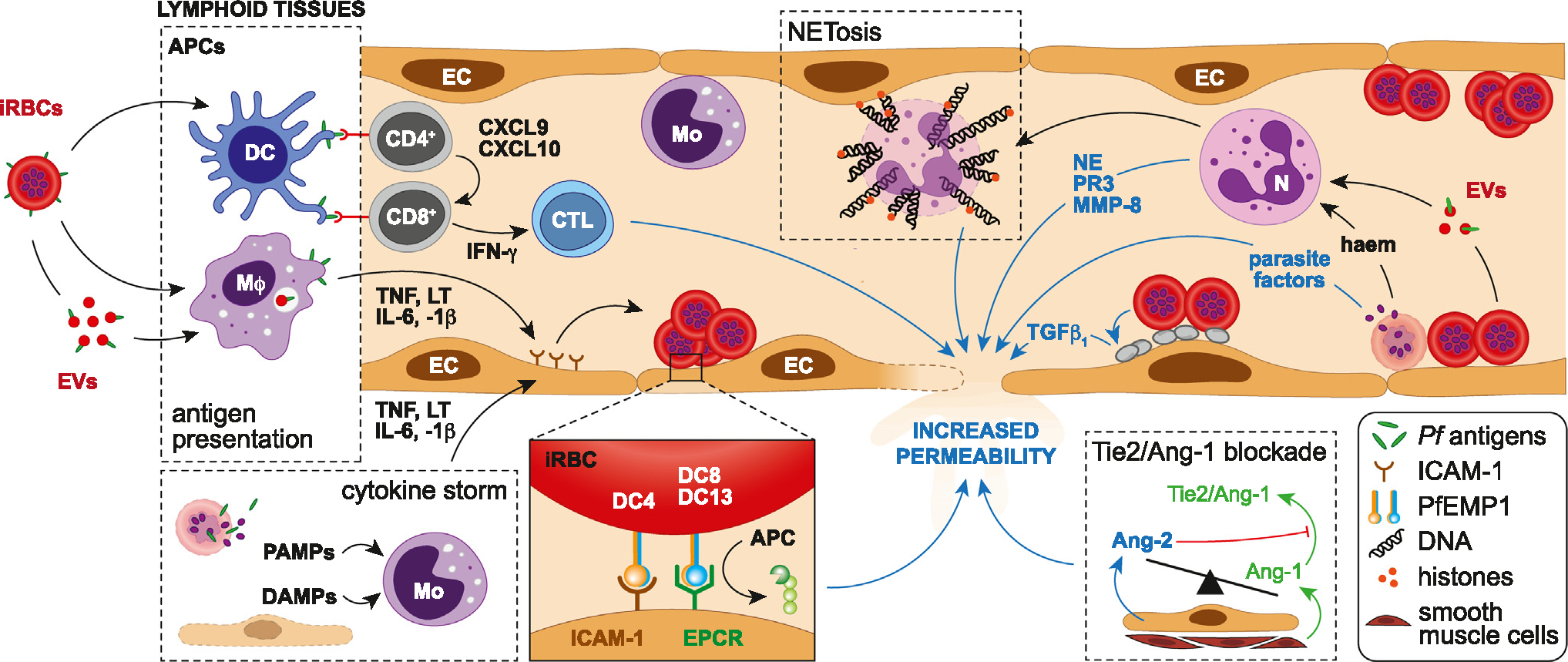
Immune response and pathogenetic mechanisms involved in blood–brain barrier (BBB) dysfunction during cerebral malaria (CM). After *Plasmodium falciparum*-infected red blood cells (iRBCs) or their antigen-carrying extracellular vesicles (EVs) are recognized and phagocytosed by dendritic cells (DCs) and macrophages (Mϕ), parasite antigens are presented to CD4^+^ T cells by their major histocompatibility class (MHC) II. Concomitantly, stimulated macrophages secrete high levels of tumour necrosis factor (TNF), lymphotoxin (LT), interleukin-6, and -1β, leading to the activation of endothelial cells (ECs) locally. Once activated by antigen-presenting cells (APCs), CD4^+^ T cells recruit CD8^+^ T cells through the release of CXCL9 and CXCL10, resulting in their activation by antigen-presenting cells via MHC I, the secretion of gamma interferon (IFN- γ) and the maturation of CD8^+^ T cells into cytotoxic T lymphocytes (CTLs). The latter then target brain endothelial cells, contributing to their destruction and BBB permeability. Pathogen-associated molecular patterns (PAMPs) released from bursting iRBCs during the intraerythrocytic cycle, and damage-associated molecular patterns (DAMPs) from injured endothelial cells, stimulate monocytes (Mo), triggering a cytokine storm and the endothelial upregulation of intercellular adhesion molecule 1 (ICAM-1), a receptor for the DC4 variant of *P. falciparum*-infected erythrocyte membrane protein 1 (PfEMP-1) that mediates sequestration. DC8/13 variants mediate the binding of iRBCs onto endothelial protein C receptor (EPCR) in the brain, abrogating the cytoprotective EPCR/activated protein C (APC) pathway. In parallel, neutrophils activated by iRBC-derived haeme and EVs release neutrophil elastase (NE), metallopeptidase-8 (MMP-8) and proteinase 3 (PRTN3), all contributing to endothelial damage. Neutrophil extracellular traps (NETs) consisting of decondensed chromatin laced with granular proteins and histones also fragilize the BBB. Parasite factors such as haem, histones, histidine-rich protein 2, and uric acid released by bursting sequestrated iRBCs can also directly increase endothelial permeability locally. The tipping of circulating angiopoietin-1/-2 (Ang-1/2) balance in favour of Ang-2 leads to the abrogation of endothelial quiescence promoted by the Ang-1-Tie-2 engagement. Lastly, platelets activated by contact with iRBCs release TGFβ1 after degranulation, which acts in synergy with TNF to induce endothelial apoptosis and alter the BBB.

Severe manifestations of falciparum malaria, including HCM, are linked to an exaggerated innate immune response to *P. falciparum* [16]. This harmful cascade of events is triggered by the innate sensing of both pathogen-associated molecular patterns and malaria damage-associated molecular patterns (PAMPs and DAMPs, respectively) by dendritic cells (DCs) and macrophages in the blood and lymphoid tissues, leading to an increased release of proinflammatory mediators, including interferon gamma (IFN-γ), tumour necrosis factor (TNF), interleukin-1 beta (IL-1β), and IL-6 [16] (Figure 2). IFN-γ can knit together many of the immune, hematological, endothelial, metabolic, and parenchymal (notably astrocytic) changes seen in both ECM and HCM [17]. The Th1 predominance created by IFN-γ overproduction can be counteracted by IL-33, which promotes the expansion of type-2 innate lymphoid cells, Th2 cytokines, and M2 anti-inflammatory macrophages [18]. IL-33 on its own can also trigger neuroinflammation and cognitive changes [19]. As endothelial cells become activated by proinflammatory cytokines, they secrete their own cytokines and chemokines, which initiate the recruitment of monocytes and T cells reported to accumulate in the cerebral microvasculature during ECM and HCM [20,21]. In turn, these leucocyte subsets release more cytokines and chemokines, further exacerbating local inflammation in the brain, dubbed ‘cytokine storm’. Over the years, a variety of cytokines and chemokines have been identified in CM pathogenesis, often acting in cascades. The role of CD4^+^ and CD8^+^ T cells in their overproduction, including IL-33, has recently been assessed [22].

Monocytes are present in HCM microvascular lesions, with younger HIV^−^ children presenting an acute disease while older HIV^+^ children present with chronic disease [23]. Locally arrested monocytes and macrophages are conspicuous in the *Plasmodium berghei* ANKA (PbA) ECM model (Figure 2). However, their role in disease, demonstrated using specific and nonspecific depletion approaches, have yielded equivocal results [6,7,24,25]. This is not surprising, as technical approaches and the disease stage targeted varies between studies, leading to variable levels of depletion, reduction in the number of other myeloid cells/macrophages, and bystander inflammation [6,24]. The role of monocytes remains unclear in ECM pathogenesis, especially at the late stages of the syndrome. They adhere sporadically to the inner vessel wall in the early stages of infection, prior to the development of clinical signs [6]. Factors as IFN-γ, TNF and lymphotoxin (LT) that promote their adherence to the endothelium via intercellular adhesion molecule-1 (ICAM-1), vascular cell adhesion protein-1 (VCAM-1), P-selectin and E-selectin, may therefore be produced far earlier than envisaged. Monocyte activity increases in the neurological stage but is confined to a few inflammatory hotspots of post-capillary venules surrounded by **perivascular macrophages** (PVMs) [26]. Caspase-1, -8, and -4 are activated in monocytes of *P. falciparum* malaria patients as well as in mice (-1, -8, -11) [27]. They promote IL-1β production, which causes fever and endotoxic shock, that can be counteracted by IL-33 treatment [28].

Neutrophils have been shown to recognize specific PfEMP1 molecules on iRBCs via ICAM-1, leading to the efficient killing of parasites associated with CM *in vitro* [29]. However, early depletion of neutrophils in mice prevented the development of ECM, suggesting a pivotal role in pathogenesis [30]. A peripheral whole-blood transcriptome study carried out in Malawian patients demonstrated an association between increased activated neutrophil transcripts and vasculopathy in HCM, despite their relative absence from the cerebral vasculature post-mortem. This suggests that by-products of neutrophil activation could mediate host organ damage and contribute to CM pathophysiology (Figure 2), as high numbers of circulating neutrophils are observed in severe malaria [31]. Such activation products include neutrophil extracellular traps (NETs), which are generated by cell death and the extrusion of decondensed chromatin decorated with microbicidal and immunostimulatory molecules. Haeme and TNF-induced NETs could therefore contribute to the immunopathogenesis of severe malaria, including CM [31]. Another indication for a pathogenic role of neutrophil activation is the local release of MMP-8, an endopeptidase stored in neutrophil intracellular granules, in the retinal tissue during paediatric HCM [32].

### Extracellular vesicles

Extracellular vesicles (EVs) are membrane-enclosed, nanosize elements produced both during homeostasis and cell activation (Box 2). Endothelial cells produce microvesicles, formerly called microparticles, which were historically the first to be found elevated in the plasma of patients with HCM, but not severe malarial anaemia [33]. Subsequently, EVs from other cellular sources, including iRBCs, have been found to play a role in both ECM and HCM [34,35]. EVs secreted from iRBCs comprise of diverse cargo (Box 1); those derived from RBCs infected with more mature parasite stages can activate neutrophils and macrophages to release proinflammatory cytokines as well as alter endothelial barrier function [36]. EVs that are secreted from earlier stages (rings) contain PfEMP1 (Figure 1). Human monocytes stimulated with these have an altered transcriptome and release low levels of inflammatory cytokines [37]. Elevated EV levels are associated with disease severity [38], but whether different *P. falciparum* strains differ in their ability to generate EVs remains unevaluated. Mature RBCs lack the appropriate cellular machinery. The exomembranous trafficking system established in the RBC by the parasite, as well as components of the parasite’s endosomal sorting complex required for transport (ESCRT), have been implicated in EV formation [39]. Thus, any mechanism that influences the synthesis of, or deposition of cargo into, or trafficking of, EVs may determine susceptibility to ECM.

### Disruption of the BBB

The BBB is a distinctive interface that tightly regulates the passage of ions, molecules, and cells between the blood and the brain [40]. Largely determined by endothelial cells, its properties are induced and maintained by pivotal interplays with mural, immune, glial, and neural cells within the neurovascular unit [41]. BBB disruption has been reported in both ECM and HCM [42]. In HCM, it results from multifarious factors, including the binding of iRBCs to the endothelial cell protein C receptor (EPCR) in cerebral microvessels, via PfEMP1 domain cassettes [43] (Figure 2). This prevents interactions between activated protein C (APC) and EPCR that normally promote cytoprotective effects on the brain endothelium. Endothelial activation, secretion of proinflammatory cytokines, shedding of EPCR, and activation of the coagulation pathway ensue. Ultimately, these events lead to barrier property loss, vascular leakage, and brain swelling in paediatric HCM [14,15]. While there are multiple potential causes of brain swelling during HCM, including an increase in parasite biomass, there is evidence of cytotoxic and vasogenic oedema in paediatric and adult CM [14] (Table 1). A recent case series suggested that these may develop sequentially [44]. Cross-site comparisons of factors associated with increased brain volume between patients from Malawi and India identified high parasite biomass, elevated transcript abundance of multiple subsets of EPCR-binding *P. falciparum*, and low platelet counts as common determinants across all age groups [45]. *In vitro* inhibition of the protease-activated receptor 1 (PAR1) to shut down potentially deleterious pathways triggered by the loss of modulatory APC-activated EPCR failed to reverse the iRBC lysate-induced disruption of barrier function, implicating a PAR1-independent mechanism [46].

The loss of BBB integrity can also be induced by *P. falciparum*-derived factors released upon iRBC rupture (Box 1) [47–49]. This effect is likely to be focal, in areas of intense sequestration in close proximity to endothelial cells, and differs from cytokine activation and damage at the transcriptomic and cellular levels [50,51].

Through their engagement with endothelial receptor Tie-2, angiopoietin (Ang)-1 and Ang-2 finely regulate endothelial cell function and vascular integrity, with opposite effects [52] (Figure 2). Ang-1 inhibits vascular permeability induced by inflammatory cytokines and attenuates pathological responses, while Ang-2 inhibits these effects [53]. The Ang/Tie-2 system plays a pivotal role in the development of CM by promoting endothelial dysregulation. In paediatric HCM, decreased levels of plasma Ang-1 and elevated levels of Ang-2 are associated with retinopathies and mortality [54]. Ang-2 exerts a permissive role on endothelial TNF activation, acting as a switch for vascular responsiveness and potentially exacerbating vascular leaks in CM. These effects can be reversed, and in mice the administration of Ang-1 at a late stage of PbA infection reinforced the BBB and improved survival [55]. In parallel, angiotensin II, the principal effector hormone of the renin–angiotensin system (RAS) has been shown to upregulate Ang-2 and decrease the Ang-1/Ang-2 ratio in endothelial cells. Gene polymorphism analyses of angiotensin-related enzymes indicated that elevated levels of angiotensin II confer reduced susceptibility to HCM in Indian patients [56]. The modulation of angiotensin II receptors protects against ECM, leading to reduced cerebral haemorrhages, increased survival in treated animals [57], and inhibitory effects on pathogenic *Plasmodium*-specific CD8^+^ T cells [58], suggesting that Ang–Tie-2- or RAS-based interventions could be used as adjunctive therapies for CM.

The role of CD8^+^ T cells in BBB impairment through the targeting of endothelial cells has long been thought to play a pivotal role in ECM [59]. Contrary to CD4^+^ T cells, CD8^+^ T cells act late and in brain microvessels [60], recognizing parasite-derived epitopes presented by endothelial cells. In ECM, disruption of BBB tight-junction proteins by antigen-specific CD8^+^ T cells occurs through a non-apoptotic, perforin-dependent mechanism [61]. BBB disruption in animals is not a result of endothelial damage, rather controlled opening of the inter-endothelial junctions [62]. In a 3D spheroid model that mimics human BBB, iRBCs were taken up by human brain endothelial cells in an ICAM-1-dependent manner, with endothelium ingress and swelling, resulting in BBB breakdown [63]. These findings are consistent with many historical studies that found no evidence of endothelial injury but only subtle changes to the BBB in human post-mortem specimens.

### New findings and hypotheses on the role of adaptive immunity

Over the past decade, pivotal new findings have contributed to the emergence of a role for different components of the adaptive immune response in the development of CM. These reports are described in the following sections and put into perspective in the context of both ECM and HCM pathogenesis, leading to new mechanistic hypotheses at the vascular and perivascular space level.

#### (i) Perivascular space and CD8^+^ T cells

**Figure 3.**
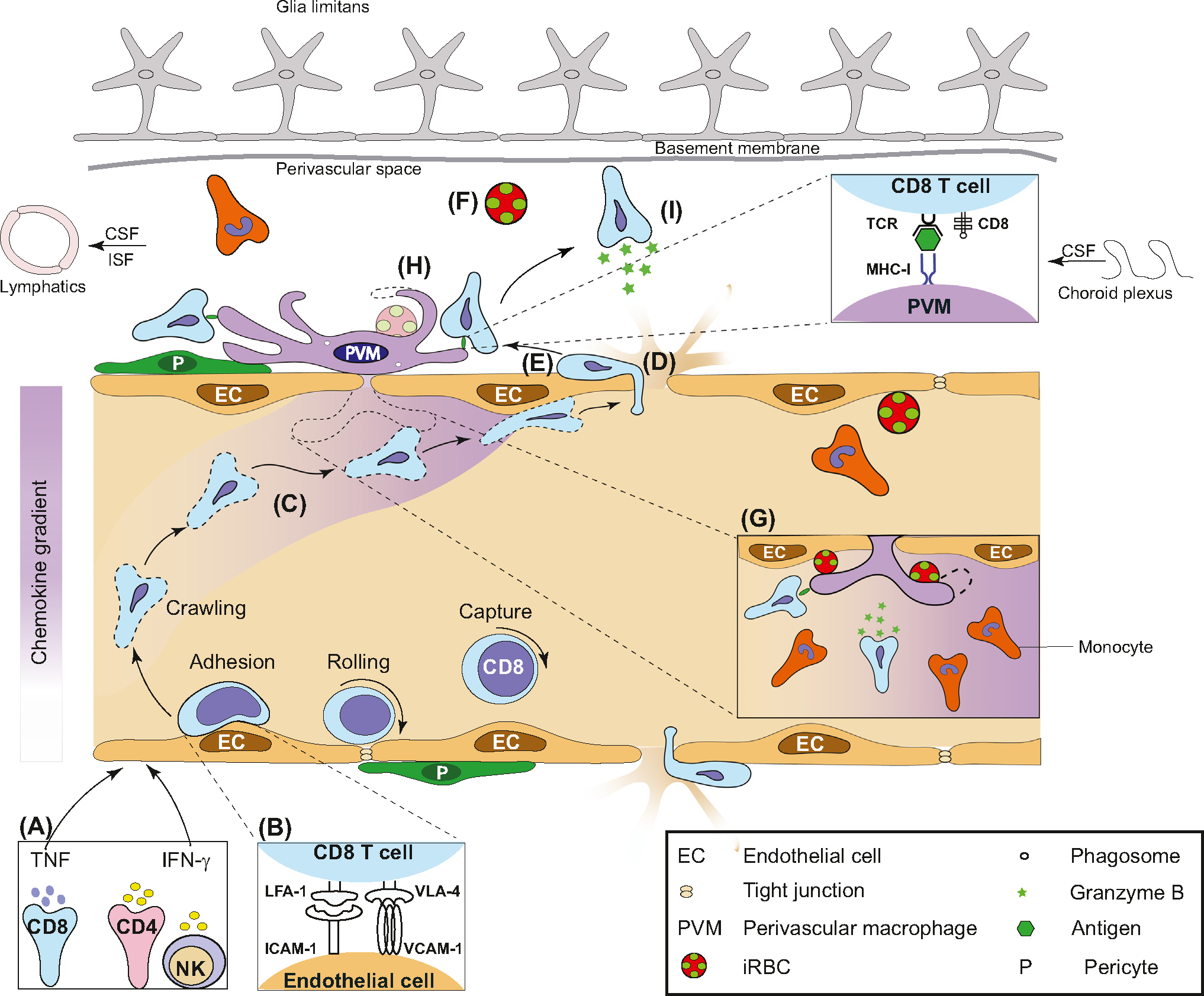
Perivascular myeloid cells create a niche for immune cell recruitment, activation, and effector function. A hypothetical model illustrating the role of perivascular macrophage (PVM) and CD8^+^ T cells in the development of experimental cerebral malaria (ECM). (A) Following *Plasmodium* infection, circulating immune cells produce proinflammatory cytokines interferon-gamma (IFN-γ) and tumour necrosis factor (TNF), which upregulate intercellular adhesion molecule-1 (ICAM-1), vascular cell adhesion molecule-1 (VCAM-1) and MHC-I on the surface of endothelial cells. (B,C) Leucocyte function-associated antigen-1 (LFA-1) and very-late antigen-4 (VLA-4) promote, and CXC-chemokine ligand 9/10 stabilize, CD8^+^ T cell adherence to the endothelium. (D) Adherence of infected red blood cells (iRBC), platelets, and endothelial vesicles lead to permeability of tight junctions. (E) T cells extravasate and crawl along the abluminal wall. (F) iRBC/parasites leak into the perivascular space. (G) PVMs lie under the basement membrane directly adjoining the endothelium alongside pericytes. PVMs phagocytose iRBCs directly from the vessel lumen. They produce chemokines for the recruitment of CD8^+^ T cells and monocytes. (H) PVMs create inflammation hotspots characterized by clustering, amoeboid appearance, phagocytosis of iRBCs in the perivascular space and MHC-I antigen presentation to CD8^+^ T cells. (I) Cytotoxic T cells release granzyme B, leading to clinical disease. Abbreviations: CSF, Cerebrospinal fluid; ISF, Interstitial fluid; NK, Natural killer cell; TCR, T cell receptor.

T cell entry into the perivascular space due to BBB breach is a key event in many infectious and autoimmune diseases [40,64]. In its simplest form, the BBB can be distinguished into two types. One type surrounds capillaries, is formed by a single layer of endothelial cells abutted by a glia limitans layer of astrocyte end feet, and is supported by a basement membrane. The second type surrounds post-capillary venules, is similar in structure, with the two layers separated by the perivascular space [40] (Figure 3). Intravital imaging studies in experimental autoimmune encephalomyelitis (EAE), an animal model for multiple sclerosis, have adeptly captured the events that unfold when T cells that promote inflammation initially gain entry [64]. T cell infiltration through the tight junctions of the venular endothelium is regulated by resident antigen-presenting cells (APCs) in the perivascular space via an antigen-dependent mechanism [65,66]. These findings are likely relevant in CM, where a similar line of enquiry is currently followed. Leucocyte infiltration into the perivascular space during CM is a distinctive feature of the venous vasculature in both mice and humans [7,21] (Figure 3). Post-mortem analyses in paediatric patients from Malawi demonstrated an infiltration of CD8^+^ T cells into the leptomeninges, pial vessels, subarachnoid space and choroid plexus [67]. Further post-mortem sample analyses showed for the first time a similar pattern of CD3^+^CD8^+^ T cell accumulation in ECM and HCM [21,68], dispelling the long-held view that this phenomenon was solely associated with ECM [69]. **Granzyme B**-expressing CD8^+^ T cells were found in close contact with CD31^+^ endothelium lining the luminal wall of the venous vasculature, as well as the abluminal wall in the perivascular space in paediatric HCM [21]. The perivascular space appears to increase in size during ECM and HCM. This cerebrovascular engagement of CD8^+^ T cells in children who died of CM was exacerbated by HIV coinfection. Long-lived cells such as PVMs, microglia, and astrocytes act as a reservoir for latent HIV, but how this might impact on CM pathogenesis remains unknown. Additionally, many activated Iba1^+^CD68^+^ macrophages in children who died of CM had localized both luminally and abluminally to the same sites where CD8^+^ T cells had released granzyme B, indicative of antigen recognition [21]. Expression of Iba1^+^ and CD68^+^ is a shared phenotypic feature of macrophage subsets, including PVMs, although this was not investigated. Recently, studies used a multiphoton microscopy approach to show that PVMs create a ‘vascular niche’ for CD8^+^ T cell arrest, which may kick-start the inflammatory cascade leading to BBB alteration and neuropathology in ECM [26,70] (Figure 3). Myeloid cells clustering around the vasculature, activation of CD8^+^ T cells and pathogen clearance via the recognition of peptides presented by H2K^b^ or H2D^b^ have been reported in ECM [26,71]. Repeated interactions of CD8^+^ T cells at PVM sites can lead to the release of cytotoxic mediators’ perforin and granzyme B, which would accumulate at inter-endothelial junctions over time and lead to BBB disruption in CM (Figure 3).

#### (ii) Perivascular myeloid cells

Perivascular myeloid cells, including PVMs, are a unique subset of immune cells that contribute to inflammation and cerebrovascular dysfunction (Figure 3). The strategic localization of PVMs beneath the endothelium in the perivascular space make them prime candidates for immunosurveillance [26]. PVMs are ideally positioned to phagocytose harmful pathogens that cross the vasculature into tissues, and are particularly abundant in the leptomeninges and subarachnoid space compared to the parenchyma. They become activated soon after pathogen entry and efficiently co-ordinate the innate and adaptive immune response [26]. Thus far, underappreciated is the role of perivascular myeloid cells in continually surveying the abluminal wall with motile process extensions during neuroinflammation. In this context, the engulfment of PbA in the perivascular space by PVMs is highly relevant [71] (Figure 3). Phagocytosis by intravascular macrophages directly from systemic circulation was first reported in 1883, with cells protruding into the vascular lumen. Similarly, PVMs have access to iRBCs in the perivascular space in the late stages of CM, when BBB disruption occurs [7]. However, it is unclear if and how PVMs sense phosphatidylserine ‘eat me’ signals and phagocytose pathogens in the early stages while localized beneath the impervious endothelial barrier [72]. Transport of antigens via an independent route – such as through the choroid plexus to the perivascular space – may be involved [73]. Of note, microglia extending processes through the intercellular junctions of the endothelium into the vascular lumen of the brain has been reported [74]. These findings provide a lead for investigating whether, and how, iRBCs may be phagocytosed by PVMs directly from the vascular lumen in the absence of a BBB breach during early-stage disease (Figure 3).

The glymphatic system, an extensive meningeal lymphatic vessel network, provides a pathway of drainage from the cranium. Tracers injected into the meningeal lymphatic vessels drained into the deep cervical lymph nodes (DCLNs) [9]. As iRBCs or their products accumulate in the perivascular space, the aforementioned studies provide compelling grounds for investigating whether *Plasmodium* antigens in the CNS can access DCLNs through the cervical lymphatics [7,9], either contributing to the deleterious immune response in CM, or tuning it down [9].

Extravasated T cells arrest and form long-term cognate interactions with CX3CR1-bearing APCs [7,75] (Figure 3), dispelling the notion that T cell activity is confined to the vascular lumen during ECM. Despite the compelling evidence of monocyte/macrophage involvement in their studies (see earlier text), Riggle *et al*. subscribed to the long-held view that *P. falciparum* antigen was being presented to T cells by the endothelium [21]. There is evidence, based on *in vitro* and *ex vivo* studies, that the endothelium can acquire and cross-present *P. falciparum* and PbA antigens [76]. However, our understanding of the structure of the endothelium has undergone a significant makeover in recent years. Seminal studies have shown that process extensions of macrophages localize to inter-endothelial junctions of blood vessels and bridge neighbouring tip cells during embryogenesis [77]. Moreover, PVMs play an important role in the maintenance of inter-endothelial junctions and in limiting vessel permeability in the steady state [78]. It is conceivable that, following uptake of iRBCs, PVMs in their activated state can promote leaky tight junctions, and process and present *Plasmodium* antigen to CD8^+^ T cells. In this context, PVMs and vascular endothelial cells are integral parts of a single anatomical and functional unit that scavenges particulate matter from renal blood vessels and monitors trans-endothelial transport [79]. This interdependent, reciprocal vascular unit supports angiogenesis, macrophage differentiation, and the integrity of endothelial cell junctions [80]. Studies assessing the role of the PVM–endothelium unit are warranted in CM.

An important question that remains unanswered is the mechanism(s) that underlies the directed migration of antigen-specific (not antigen-ignorant) T cells towards focal areas of PVM localization along the luminal wall [26]. Of relevance, T cells transmigrating across the inner vessel wall in a highly constrained manner at APC sites in response to antigen recognition has been reported in EAE [81]. Such a mechanism could explain how focal chemokine gradients – created by PVMs at inter-endothelial junctions – may lead to antigen-specific T cell recruitment in the absence of a BBB breach [82]. Nuclear lobes of leucocytes can squeeze through junctions without rupturing endothelial stress fibres [83]. As a next step, process extensions of PVMs could provide a structural scaffold for guiding T cells along the abluminal wall. Regardless of what the mechanism might be in the early stage, disruption of endothelial tight junctions in later stages of infection will no doubt provide an access route for T cells to preferentially extravasate at circumscribed locations adjacent to PVMs.

#### (iii) Antigen presentation: parasite, endothelium, and PVMs

CD8^+^ T cells specific for PbA antigens migrate from the spleen to the brain, guided by chemokines such as CXCR3 [84]. CD8^+^ T cells must recognize antigen once again in the brain before killing can occur [85]; the latter happens within minutes once T cells receive a ‘license’ from an APC. Indeed, endothelial cells and parenchymal brain macrophages expressed higher levels of MHC class I during ECM [71]. The additional stimulus in the form of parasitic antigen to brain-infiltrated CD8^+^ T cells is provided by an APC, endothelium, or both, at which time cytotoxic enzymes are released. Mice deficient in granzyme B and perforin are protected against ECM [85]. Adoptively transferred wild-type CD8^+^ T cells could reverse this effect but only when a critical threshold of parasitic antigen was presented in the brain. These observations demonstrate the requirement for T cells to receive local antigen presentation signalling.

With respect to parasite antigens, iRBCs release an array of parasite factors, including waste products and mature merozoites upon completion of the cell cycle when the RBC bursts and merozoites egress (Box 1). In addition, iRBCs also secrete EVs that contain diverse cargo, including proteins, nucleic acids, regulatory **microRNAs** and lipids [86]. Because of iRBC sequestration via PfEMP1, these parasite products concentrate at the endothelium where they can directly act on endothelial cells through either activation of Toll-like receptors or inflammasomes. Thus, parasite products not previously considered to serve as parasite antigens may in fact contribute to HCM via an array of mechanisms (Box 1) [16,87]. Additionally, other proinflammatory cytokines, liberated in response to parasite factors, can induce endothelial activation, including the induction of iRBC receptor ICAM-1. This process, which is regulated by rapid nuclear translocation of p65 NFκB subunit, in turn would augment even further sequestration of iRBCs either directly or via platelet bridging [88], leading to microvascular obstruction (Figure 1), impairment of the endothelial barrier (Figure 2), and ultimately resulting in vascular leakage and microthrombi [89]. While the source of antigen cross-presented by brain endothelial cells has been demonstrated to be primarily of merozoite origin in ECM [76], a deeper understanding of how iRBC antigens, in addition to PfEMP1, are presented to the PVM-endothelium unit, and lead to inflammation, is critical for understanding how HCM arises and may inform strategies for adjunct therapy.

**Figure 4.**
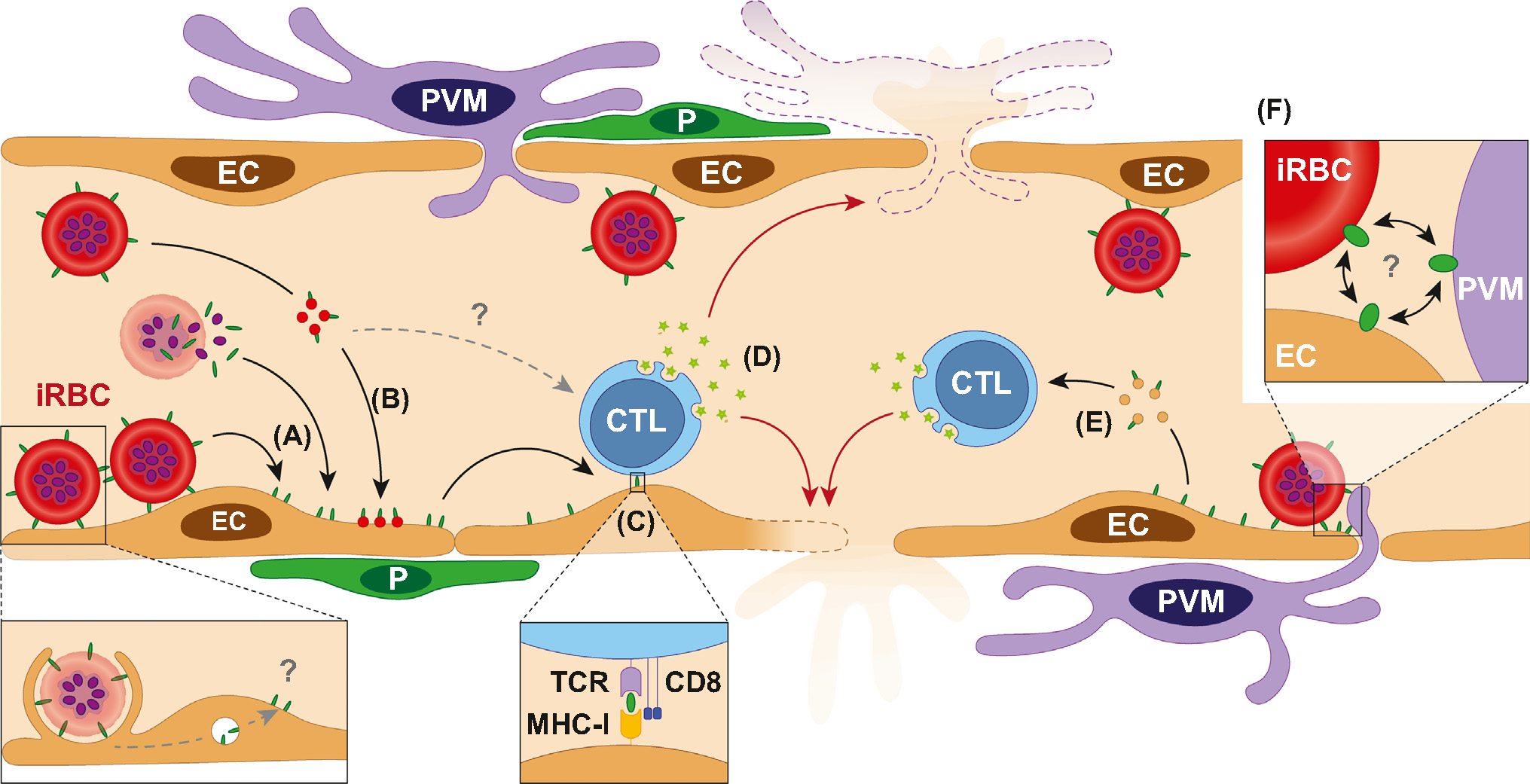
Proposed role for the immune targeting of antigen-presenting cells (APCs) in the blood–brain barrier (BBB) breakdown during cerebral malaria (CM). (A) *P. falciparum*-infected red blood cells (iRBCs) can transfer parasite antigens to the surface of endothelial cells (ECs) through either direct membrane contact or release of antigen upon schizont rupture. (B) iRBCs produce antigen-carrying extracellular vesicles (EVs) that bind to and/or fuse with the endothelial surface, thereby transferring parasite antigens to ECs. Erythrophagocytosis of iRBCs by ECs contribute to this antigen-presentation pathway (lower dash gray arrow, ‘?’). (C) Antigens are presented by ECs through their MHC-I. (D) Cytotoxic T lymphocytes (CTLs) engage with ECs and perivascular macrophages (PVMs) via their T cell receptor (TCR), leading to the release of granzyme B, targeting of ECs, and BBB permeability, ultimately resulting in vasogenic edema. Antigen-carrying EVs shed by iRBCs may contribute to CTL activation (upper dash gray arrow, ‘?’). (E) Parasite antigens are presented by endothelial EVs. (F) The physical proximity between ECs, PVMs, and iRBCs at sites of dense sequestration may increase their interactions, further exacerbating antigen transfer and presentation, leading to foci of BBB disruption and cerebral microhaemorrhage reported both in experimental and human CM. Abbreviation: P. pericyte.

Due to their physical location at the interface between blood and surrounding brain tissue, endothelial cells are exposed early to blood-borne microbial components released in the circulation (Figure 4). They express a wide range of pattern-recognition receptors [90], allowing brain endothelial cells to act as early innate immune responders in *P. falciparum* infection and potentially contributing to CM pathogenesis [91]. Some of the aforementioned mechanisms (see Disruption of the BBB) are further amplified by the capacity of endothelial cells to act as semi-professional APCs [92], or to have macrophage-like functions [93], or uptake and present parasitic antigens during ECM [71,94,95] (Figure 4). Using luciferase-expressing *Plasmodium* strains PbAluc and PyYMluc, the ability of endothelial cells to cross-present a specific epitope (SQLLNAKYL) was shown to be reflective of the aptitude of parasites to sequester in the brain and cause ECM [95]. Although PbA sequestration does not lead to vessel occlusion, even a modest accumulation of iRBCs within the vasculature and the perivascular space may provide a localized source of antigen for cross-presentation by endothelial cells, leading to CD8^+^ T cell recruitment, proliferation, and activation [96]. CD8^+^ T cells can home onto their cognate antigen on or behind the BBB, a phenomenon dependent on luminal expression of MHC class I by cerebral endothelium [97]. iRBCs and/or parasite antigens can be accessed from the cerebral microvasculature through endothelial erythrophagocytosis [63], or trogocytosis, where antigens are extracted from the surface of iRBCs [98] (Figure 4). Both of these processes can lead to antigen presentation. An additional conceptual link between APC function, antigen presentation, and CM pathogenesis is provided by CXCL10 [99], which stabilizes T cell–brain endothelial cell adhesion during ECM and is a serum biomarker that predicted CM mortality in Ghanaian children [100,101].

Imaging applications in other models have begun to provide insights into when and where antigen presentation takes place *in vivo*, and for how long [66]. CD4^+^ T cells entering the brain have been shown to arrest along the vascular endothelium, where polarization of T cell receptor (TCR) and adhesion molecules occur [81], suggesting that these fixed points are sites of immune synapses. Following contact, antigen-specific T cells penetrate the CNS during EAE [64]. In peripheral lymphoid organs, the type of contact that occurs between APCs and T cells determines the functional outcome, with short contacts inducing tolerance and long contact activating T cells [102]. In this context, studies showed that PbA-primed CD8^+^ T cells first enter the brain in the early stage and arrest to inner vessel ‘hotspots’ either directly adjacent to, or in the near vicinity of, PVMs [26], the identity of which was established using reporter mice [103]. PbA-specific CD8^+^ T cells arresting to the endothelial wall and becoming activated in a cognate peptide-MHC class I-dependent manner during ECM have been reported [71]; however this study utilized non-reporter Kb^−/−^Db^−/−^ mice and so, whether PVMs, additionally to endothelium, had a role in antigen presentation could not be ascertained [26]. Nevertheless, all the aforementioned studies collectively suggest that only CD8^+^ T cells that recognize cognate antigen are retained in the brain. T cells became aggressive in the late stages of ECM, their interaction rates increased at hotspots, potentially signifying their search for an exit into the perivascular space [26].

## Concluding remarks

The development of new adjunct therapies in CM has proven expensive, difficult, and disappointing to date, with no clear clinical benefit in sight for patients [104]. While the repurposing of existing drugs, such as losartan [57], has shown promising results in ECM, their assessments in patients with CM remain challenging owing to the large sample sizes required to detect clinically meaningful differences in mortality and resulting high costs and logistical hurdles. More than ever, innovative, collaborative, and transdisciplinary approaches using clinical samples, *in vitro* and *ex* vivo models, and animal models of the disease, will be pivotal to allow global advances in CM survival (see Outstanding questions). As a step in this direction, here we have attempted to identify areas of priority and immediate relevance for CM. Understanding the fine immune mechanisms involved in CM will likely facilitate the development of new host-directed therapies that target specific molecules, pathways, or networks, offering exciting new opportunities for prevention and treatment.

Future studies must distinguish the phenotype of perivascular APCs abutting the luminal and abluminal wall of the cerebral vasculature in falciparum malaria. The localization and function of perivascular APCs within the anatomical unit of the BBB needs further examination. The potential for brain APCs to serve as a reservoir for latent HIV and modulate the immune response could help to explain why HIV is a risk factor for CM and death from CM. New insights into the mechanistic role of APCs in producing chemoattractants and promoting focal areas of inflammation can be successfully exploited for targeted treatment of inflammation. T cell migration interference can be executed at different stages, including recruitment, antigen recognition, activation, extravasation, effector function, and tissue damage. Whether iRBCs or their products reach the perivascular space due to BBB disruption (via the choroid plexus route or leakage of luminal contents) or in the absence of BBB disruption (via direct engulfment of luminal parasites/parasitic antigens or an independent route, such as the glymphatics draining to the DCLN), needs serious consideration (see Outstanding questions). Continued investment in high-resolution molecular techniques will be needed to examine whether antigen presentation is a prerequisite for immune cell activation, BBB disruption and CM outcome.

## Figures and Tables

**Table 1.** Comparison offeatures of CM in humans and mice^a^

	Children^b^	Adults^c^	ECM^d^	Refs
Neuroimaging features (acute stage)				
Brain swelling	Decreases with age		Yes	[14,15,45,105]
Cytotoxic oedema	Yes^e^	Yes^f^	Yes	[14,106]
Vasogenic oedema	Yes	Yes	Yes	[105–107]
Vascular congestion	Yes	Yes	NR	[107,108]
Microhemorrhages	Yes	Yes	Yes	[105,108]
PRES-like features	Yes	Yes	NR	[107,108]
Vasospasm/vasoconstriction	Yes	Yes	Yes	[109]
Behavioural changes				
Convulsions	Yes	Yes	Yes	[3,110,111]
Paralysis, seizures	Yes	Yes	Yes	[3,110,111]
Histopathological features in the brain				
Astrocyte morphological changes	DG	DG	Yes	[112,113]
Microglial/macrophage morphological changes	Yes	Yes	Yes	[113,114]
iRBC accumulation in vessels	Yes	Yes	Yes	[12,13]
Knobs on iRBC	Yes	Yes	No	[115]
Mononuclear leukocyte adhesion to endothelium	Yes	Yes	Yes	[6,7,23,67,111]
Presence of platelets in microvessels	Yes	NR	Yes	[23,110]
Neuronal damage	NR	Yes	Yes	[113,116,117]
Focal demyelination	NR	Yes	Yes	[113,117]
Expression of molecules in brain or retina				
Upregulation of ICAM-1 on endothelium	Yes	Yes	Yes	[110,118]
Overexpression of MHC	Class I/II	NR	Class I/II	[71,110]
TNF mRNA and protein expression	Yes	Sometimes	Yes	[110,111]
TNFR2	Yes	NR	Yes	[110,111]
Histopathological features in the retina				
Haemorrhages	Yes	Controversial^g^	Yes	[107,119]
Blood-brain barrier function				
Increased vascular permeability	Yes	Controversial	Yes	[3,111]
Transcellular disruption	NR	NR	Yes	[105]
Biochemical changes in brain				
Increased lactate	Yes	Yes	Yes	[111]
Increased kynurenine pathway metabolites	Yes	Yes	Yes	[111]

a Abbreviations: CM, cerebral malaria; DG, Durck's granuloma (a gliotic lesion containing microglia and astrocytes); ECM, experimental cerebral malaria; ICAM-1, intercellular adhesion molecule-1; iRBC, infected red blood cell; MHC, major histocompatibility complex; NR, not reported; PRES, posterior reversible encephalopathy syndrome; TNF, tumour necrosis factor; TNFR2, tumour necrosis factor receptor-2.

b Children from sub-Saharan Africa and India.

c Adults from Thailand, Vietnam, Bangladesh, India, Colombia, or returning travellers.

d *Plasmodium berghei* ANKA in CBA, A/J or C57 mice.

e In the white matter, reversible in non-fatal cases.

f In the basal ganglia, reversible in non-fatal cases; generalized in fatal cases.

g Retinopathies are associated with CM in African children, but the same findings were not consistently reported in adult patients from India and Bangladesh.

## Box 2. Specific pathogenetic mechanisms of EVs in CM

- High levels of endothelial EVs were originally found to correlate specifically with CM as they were not elevated in patients with severe malarial anemia, indicating that endotheliitis underpins CM development.
- High levels of EVs from all circulating cell types indicated increased cellular activation in the vascular compartment.
- Conceptually, dramatic changes observed in the properties of EVs suggest a crucial role in CM. They are induced by TNF, elevated in CM, and their inhibition prevents CM pathology. They can transfer antigens from platelets to endothelial surfaces, activate macrophages, alter permeability, and trigger signalling changes.
- Adoptive transfer of EVs from mice with CM triggers CM-like pathology in naïve recipients.
- As EVs participate in antigen presentation, express accessory molecules, and modulate T cells, they can influence susceptibility versus disease tolerance to CM, by favouring trained immunity or trained tolerance.
- Specific aspects of EVs from infected red blood cells (iRBCs): see Box 1.
- EVs released by iRBCs contain parasitic small RNAs and genomic DNA, and phagocytosis by monocytes triggers STING-dependent sensing in the cytosol. This DNA-sensing pathway may be an important decoy mechanism to promote malaria parasite virulence.

Much remains to be known about the importance of the EV corona in CM. The corona obviates the need for EV to fuse with recipient cell membranes, so that major immunomodulatory changes can be triggered without being internalized.

## Outstanding questions

What are the fine, local immune mechanisms involved in the development of CM? Elucidating these through multidisciplinary approaches and combining clinical investigations, laboratory and animal models of the disease will likely identify new therapeutic avenues.

What is the localization, phenotype, and function of antigen-presenting cells in the cerebral vascular and perivascular space in *Plasmodium falciparum* infection? Can these cells be targeted to dampen focal inflammation and increase survival in CM?

Can iRBCs and their by-products reach the perivascular space during CM, irrespective of the BBB breakdown? What are the consequences of this transfer in terms of local inflammation and neuronal damage? Is this linked to long-term neurocognitive sequelae in survivors? Further mechanistic studies are warranted to decipher the effects of focal inflammation and neurocognitive impairment.

Which chemoattractants, produced by perivascular APCs, promote focal areas of inflammation at the BBB? Can this be exploited for targeted treatment of inflammation in CM?

What immune genetic variants and environmental factors drive susceptibility or resistance to CM? In particular, what are the links between autoimmunity and malaria susceptibility?

## Highlights

New evidence has widened the importance of innate immunity in the development of cerebral malaria (CM), including the role of neutrophils, their extracellular traps, and their contribution to the breakdown of the blood–brain barrier (BBB).

In parallel, reports show that brain perivascular macrophages and endothelial cells can act as antigen-presenting cells for CD8^+^ T cells, and their collateral targeting may contribute to disruption of the BBB.

The vascular and perivascular spaces are likely to be the scene of key pathogenic events in cerebral malaria, and interactions between immune cells, endothelium, and infected red blood cells need to be carefully examined in this context.

## Glossary

Central nervous system (CNS): the brain and spinal cord; the CNS plays a pivotal role in processing and coordinating information throughout the body.
Cytoadherence: the binding of infected erythrocytes to endothelial cells in different organs as a mechanism to evade host immune removal; it leads to sequestration.
Endothelium: a thin cell monolayer that lines blood vessels and releases substances that control vascular contraction and relaxation, blood clotting, and immune functions.
Glymphatic (system): a recently discovered macroscopic waste-clearance system that utilizes perivascular channels formed by astroglial cells to eliminate soluble proteins and metabolites from the CNS.
Granzyme B: a serine protease found in the granules of natural killer cells and cytotoxic T cells; it acts as a caspase-like enzyme, contributing to apoptosis (programmed cell death) in virus-infected and tumour cells.
MicroRNAs: small, single-stranded, noncoding RNA molecules, typically 21–23 nucleotides long; they are involved in the regulation of gene expression.
Myeloid cells: a group of white blood cells – including granulocytes, monocytes, macrophages, and dendritic cells – that circulate through the blood and lymphatic system and are rapidly recruited to sites of tissue damage and infection via various chemokine receptors.
Perivascular macrophages: specialized resident macrophages located in the perivascular space around blood vessels in the brain; they play crucial roles in maintaining vascular homeostasis and immune responses.
Tonsillar herniation: downward displacement of the cerebellar tonsils through the foramen magnum, a large oval-shaped opening in the occipital bone that transmits the spinal cord.
